# Maternal experience of intimate partner violence is associated with suboptimal breastfeeding practices in Malawi, Tanzania, and Zambia: insights from a DHS analysis

**DOI:** 10.1186/s13006-021-00365-5

**Published:** 2021-02-18

**Authors:** Christine N. Walters, Hasina Rakotomanana, Joel J. Komakech, Barbara J. Stoecker

**Affiliations:** Department of Nutritional Sciences, Oklahoma State University, Stillwater, OK USA

**Keywords:** Breastfeeding, Intimate partner violence, Malawi, Tanzania, Zambia

## Abstract

**Background:**

Despite widespread suboptimal breastfeeding practices and maternal experiences of intimate partner violence (IPV), the association of IPV and breastfeeding practices remains unclear. This study investigated the associations between maternal experience of physical, sexual, emotional violence, and controlling behaviors with suboptimal breastfeeding practices in Malawi, Tanzania, and Zambia.

**Methods:**

Data included mother-infant dyads from the most recent Demographic and Health Surveys for Malawi (*n* = 1878), Tanzania (*n* = 3184), and Zambia (*n* = 3879). Intimate partner violence (physical, sexual, and emotional) was measured using the Revised Conflict Tactics Scale. Breastfeeding practices were early initiation of breastfeeding (within 1 h of birth), exclusive breastfeeding (in previous 24 h), and continued breastfeeding. Associations between experience of physical, sexual, or emotional violence or controlling behaviors and breastfeeding practices, as well as associations between the frequency of IPV and breastfeeding practices were assessed.

**Results:**

Many Malawian (77.6%) and Zambian (67.7%) and just over half (53.6%) of Tanzanian mothers, initiated breastfeeding within 1 h of birth. Exclusive breastfeeding was 70.6% in Zambia and 60.1% in Malawi, while 55.0% of Tanzanian mothers breastfed exclusively. Continued breastfeeding to at least 1 year was high in Malawi 92.2%, Tanzania 93.4%, and Zambia 95.0%. Most mothers reported experiences of IPV in Tanzania 79.1%, Zambia 78.9%, and Malawi 73.9%. Mothers who experienced sexual IPV were significantly more likely to delay breastfeeding (Malawi [AOR 1.55 (1.14, 2.10)]; Tanzania [AOR 1.30 (1.04, 1.62)]; and Zambia [AOR 1.28 (1.06, 1.54)]). Sexual IPV in Malawi and Zambia was associated with greater odds of not exclusively breastfeeding (Malawi [AOR 1.90 (1.05, 3.45)]; Zambia [AOR 1.75 (1.15, 2.67)]). Tanzanian mothers who experienced IPV often or sometimes were two times more likely not to breastfeed at one-year post-delivery [AOR 2.23 (1.09,4.57)].

**Conclusions:**

In the three countries investigated maternal experience of IPV was associated with suboptimal breastfeeding practices. Policies and programs targeting improved breastfeeding practices should consider screening during antenatal and postnatal care for experience of violence and support initiatives to reduce IPV.

## Background

Globally, undernutrition is linked to approximately 45% of all deaths among children under 5 years of age [1]. Optimal nutrition is critical to healthy growth and development, especially during the first 1000 days [2]. Breastmilk, the first food for infants, provides the nutritional needs for the first 6 months of life [1] and promotes optimal neurodevelopment while decreasing the incidence of infectious diseases and infant mortality [2]. Consequently, unless contraindicated, the World Health Organization (WHO) recommends that newborns be breastfed within the first hour of birth and that infants be exclusively breastfed (EBF) until they are 6 months of age. Additionally, the WHO recommends that breastfeeding be continued at least until the first birthday and up to 2 years or beyond [3].

In 2018, WHO and United Nations Children’s Fund published the Global Breastfeeding Scorecard which set targets for breastfeeding rates by 2030 of early initiation of breastfeeding at 70%, EBF at 70% and continued breastfeeding at 80% [4]. Yet, despite the known benefits and recommendations, breastfeeding rates remain below these targets in many countries, including those in sub-Saharan Africa [5]. A meta-analysis of sub-Saharan African Demographic and Health Surveys (2010–2015) found that prevalence of early initiation of breastfeeding was low in Tanzania (49%) and Zambia (57%) [5]. Previous Demographic Health Surveys also have reported the following EBF rates in Tanzania (50%), Zambia (61%), and Malawi (71%) [6–8].

In efforts to improve breastfeeding practices, researchers have sought to uncover the determinants of breastfeeding [9, 10]. While the evidence is wide-ranging on factors associated with breastfeeding practices, psychosocial factors such as maternal experience of intimate partner violence (IPV) are not well-understood. Intimate partner violence refers to the behaviors within an intimate relationship (partner or spouse) that cause psychological or emotional, physical, or sexual harm [11]. Intimate partner violence also includes controlling behaviors, such as restricting access to financial resources, medical care, or limiting social interactions [11]. Reports show that maternal experience of IPV in Malawi, Zambia, and Tanzania is common [6–8]. In the 2007 DHS survey, 47% of Zambian women reported physical IPV and 41.8% reported that their husbands displayed three or more controlling behaviors [7]. In the 2010 DHS, 25% of Malawian women reported having experienced emotional IPV and 19% reported sexual IPV [8], while 39% of Tanzanian women reported physical IPV [6].

Despite the common occurrence of IPV, impacts on breastfeeding remain unclear. However, two contradicting theories, the deficit hypothesis [12] and the compensatory hypothesis [13] have been proposed to explain a probable link between IPV and breastfeeding practices. The deficit hypothesis suggests that mothers who experience violence may have physical, psychosocial, or emotional barriers to breastfeeding, and therefore, are less likely to breastfeed [12]. Contrarily, the compensatory hypothesis proposes that maternal experience of IPV may improve breastfeeding practices as a result of the mother’s increased sensitivity to the needs of her infant [13].

Overall, most evidence across countries suggests that mothers who experience IPV are less likely to breastfeed optimally [14–19], which is consistent with the deficit hypothesis. However, one study that included Malawian, Tanzanian, and Zambian mothers reported that those who experienced IPV were more likely to have optimal breastfeeding practices [20]. Therefore, because previous findings in Malawi, Tanzania, and Zambia, unlike several other African countries [20], supported the less common compensatory hypothesis, further investigation of associations between maternal experience of IPV and breastfeeding in these three countries is needed.

Furthermore, most studies reported lifetime IPV [14–20] which may not capture differences between mothers who currently experience IPV as opposed to those who experienced IPV in the past. Women who experience either past or current IPV may both have negative effects [11], but their breastfeeding choices may differ. Perhaps women who experienced IPV in the past may have had time to receive resources or support to help mitigate the negative effects of IPV on breastfeeding, and thus align more with the compensatory hypothesis.

Additionally, data are lacking on impacts of controlling behaviors, despite these being a form of IPV [11]; most studies have focused on the associations between physical, sexual, or emotional IPV and breastfeeding [14–20]. Because controlling behaviors can make a mother less autonomous by isolating her from support systems or restricting her access to medical care, it is important to understand how this form of IPV may influence a mother’s breastfeeding practices. Previous findings in India suggested that maternal autonomy was associated with a higher likelihood of breastfeeding [21].

Therefore, the purpose of this study was to investigate the associations between physical, sexual, and emotional violence, as well as controlling behaviors, with early initiation of breastfeeding, EBF, and continued breastfeeding in Malawi, Tanzania, and Zambia. Additionally, we analyzed associations between frequency of IPV with breastfeeding practices in each country. Besides advancing the understanding of IPV and breastfeeding, the results from this study may support interventions, programs, and policies that seek to improve breastfeeding practices.

## Methods

### Data source and sampling

Data were obtained from the most recent Demographic and Health Surveys (DHS) for the following neighboring African countries of Malawi (2015–2016), Tanzania (2015–2016), and Zambia (2013–2014). Each of the country’s DHS used stratified, cluster sampling. While all women 15–49 years were eligible for interviews, only a random subsample were selected for the Revised Conflict Tactics Scale (CTS-2) [22] used to assess intimate partner violence. Our analysis for each country included women 15–49 years of age who had an infant aged between 0 and 24 months, who completed the CTS-2 scale and answered questions about breastfeeding.

**Fig. 1:**
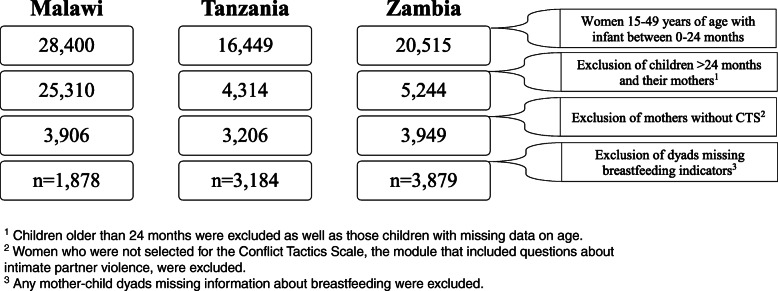
Details of sampling and exclusion of mother-child dyads from the data sets

**Fig. 2:**
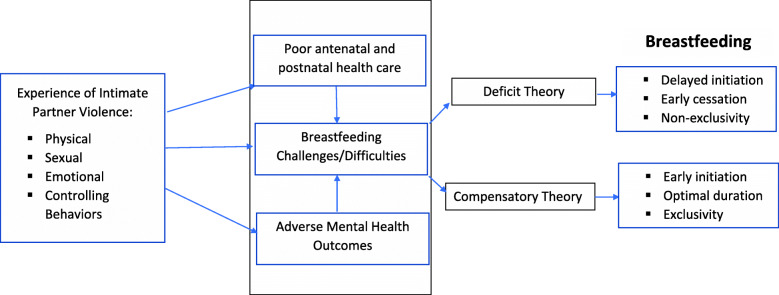
Potential pathways between maternal experience of IPV and breastfeeding

Figure 1 shows the original sample size for each country’s mother-child datasets, the exclusion criteria and the final sample sizes used for analyses. Our conceptual framework outlining the pathways between maternal experience of IPV and breastfeeding is shown in Fig. 2.

### Analyses

The three study outcomes were the WHO’s first three Infant and Young Child Feeding (IYCF) indicators: 1) early initiation of breastfeeding 2) EBF, and 3) continued breastfeeding [3]. Early initiation of breastfeeding was measured as the proportion of children born in the last 24 months who were put to the breast within 1 h of birth. Exclusive breastfeeding was measured as the proportion of infants 0–5 months of age who were fed exclusively with breastmilk in the previous 24 h. Continued breastfeeding was measured as the proportion of children 12–15 months of age who were fed breastmilk in the previous 24 h.

Using the CTS2 [22], maternal experience of physical IPV included the following nine variables: ever been pushed, shook, had something thrown at you, slapped, punched with fist or hit with something, kicked/dragged, strangled/burnt, threatened with knife/gun/other weapon, or had arm twisted/hair pulled. Emotional IPV was assessed by three variables: reported experience of humiliation by husband/partner, threatened with harm, insulted or made to feel bad. Sexual IPV included: being physically forced into unwanted sex, forced into other unwanted sexual act, or physically forced to perform sexual act. In addition to the CTS2, the DHS questionnaire also included a series of questions about whether partners ever displayed controlling behaviors within their relationship. Controlling behavior was described as: whether the husband/partner was jealous if respondent talks with other men, whether he accuses the respondent of unfaithfulness, whether he forbids respondent to meet her friends, whether he tries to limit respondent’s contact with family, whether he insists on knowing where respondent is, or whether he does not trust her with money.

Intimate partner violence exposure was measured by maternal reported experience of specific forms (physical, sexual, emotional, or controlling behaviors) and frequency (never, yes but not in the last 12 months, often/sometimes). Not having experienced any form of IPV was coded 0 and having experienced a form of IPV was coded 1. Frequency of experience of any form of IPV was coded as: 0 = never, 1 = yes but not in the last 12 months, 2 = often/sometimes. Frequency of controlling behavior was not collected and therefore could not be analyzed.

Child, maternal, and paternal characteristics for each country were computed using descriptive statistics and weighted frequencies. The weighted proportions of children meeting breastfeeding indicators and percentage of mothers who reported experience of IPV were calculated for each country. Bivariate logistic regressions were performed to determine the association between IPV exposures and each breastfeeding outcome. Variables that reached a *p* - value < 0.05 in the bivariate analyses were analyzed using multivariate logistic regression. No variables reached a *p* - value between 0.10 and 0.05 in the bivariate analyses. Multivariate analyses were adjusted for strata, cluster, and for covariates including the child’s sex, maternal age, occupation, education, literacy, exposure to media, type of delivery (Caesarean section or vaginal), delivery location, birth spacing, antenatal visits, household wealth index, and urban or rural residence [23]. Multicollinearity of explanatory variables was tested using the Variance inflation factor (VIF) and the tolerance test and results were within normal limits. Variables with a *p* - value < 0.05 were considered significant in the multivariate analyses. SAS, v. 9.4, was used for statistical analyses.

## Results

### Child, maternal, and paternal characteristics

Table 1 summarizes child, maternal, and paternal characteristics for Malawi, Tanzania, and Zambia. In all three countries, child stunting was high, in Malawi (33.8%), Tanzania (32.7%), and Zambia (36.1%). Incidence of underweight and wasting were lower but generally similar across countries. Child morbidity such as fever (36.2%) and diarrhea (36.6%) were particularly common among Malawian children.

**Table 1:** Child nutrition and health characteristics and sociodemographic and IPV data

	Malawi			Tanzania			Zambia		
	n	%	Mean (SD)	n	%	Mean (SD)	n	%	Mean (SD)
Child									
**Age (months)**	1878	–	11.9 (7.1)	3184	–	12.2 (7.1)	3879	–	12.1 (7.0)
**Sex**									
Male	949/1878	50.5		1597/3184	50.2		1973/3879	50.9	
Female	929/1878	49.5		1587/3184	49.8		1906/3879	49.1	
**Stunting**	620/1834	33.8		793/2426	32.7		1355/3758	36.1	
**Underweight**	184/1834	10.0		346/2426	14.3		484/3758	12.9	
**Wasting**	58/1834	3.2		136/2426	5.6		263/3758	7.0	
**Diarrhea (in last 2 weeks)**	511/1396	36.6		524/2426	21.6		829/3758	22.1	
**Fever (in last 2 weeks)**	505/1396	36.2		549/2426	22.6		902/3753	24.0	
Maternal									
**Age (years)**	1878	–	26.8 (6.4)	3184	–	28.2 (6.7)	3879	–	27.9 (6.5)
**Number of antenatal visits**	1835	–	3.8 (4.2)	3058	–	3.7 (4.4)	3741	–	4.2 (7.4)
**Education**									
No education	225/1878	11.9		658/3184	20.7		455/3879	11.7	
Primary education	1283/1878	68.3		2092/3184	65.7		2288/3879	59.0	
Secondary education or higher	370/1878	19.7		434/3184	13.6		1136/3879	29.3	
**Marital status**									
Married	1564/1873	83.5		2058/3184	64.6		3545/3865	91.7	
Living with partner	139/1873	7.4		859/3184	27.0		27/3865	0.7	
Widowed/divorced/separated	170/1873	9.1		267/3184	8.4		293/3865	7.6	
**Occupation**									
Not working outside the home	548/1873	29.3		615/3184	19.3		1603/3865	41.5	
Working outside the home	1325/1873	70.7		2569/3184	80.7		2262/3865	58.5	
**Wealth**									
Poorest/poorer	950/1873	50.7		787/3184	24.7		2075/3865	53.7	
Middle	371/1873	19.8		633/3184	19.9		741/3865	19.2	
Richer/richest	552/1873	29.5		1764/3184	55.4		1049/3865	27.1	
**Believed beating was justified**	299/1873	16.0		1986/3184	62.4		2070/3865	53.6	
**Fear of partner**									
Never	808/1873	43.1		1768/3184	55.5		1796/3865	46.5	
Sometimes	714/1873	38.1		919/3184	28.9		1441/3865	37.3	
Most of the time	351/1873	18.8		497/3184	15.6		628/3865	16.2	
Partner/Spouse									
**Age**	1682		32.3(9.4)	2913		35.2(9.1)	3410		33.8 (7.9)
**Education**									
No education	158/1682	9.4		412/2913	14.2		270/3758	7.2	
Primary education	953/1682	56.7		2008/2913	68.9		1640/3758	43.7	
Secondary education or higher	571/1682	33.9		493/2913	16.9		1848/3758	49.1	
**Occupation**									
Not working	140/1682	8.3		26/2913	0.9		59/3758	1.6	
Technical and managerial	245/1682	14.6		140/2913	4.8		808/3758	21.5	
Agriculture	665/1682	39.5		1652/2913	56.7		2116/3758	56.3	
Services, manual labor, and other	632/1682	37.6		1095/2913	37.6		775/3758	20.6	
**Physically hurt by wife/partner**	65/1682	3.9		53/2913	1.8		236/3757	6.3	

For each country, primary education was the highest level achieved for more than half of the mothers (Malawi 68.3%; Tanzania 65.7%; Zambia 59.0%). In all three countries, the majority of mothers were working outside the home (Malawi 70.7%; Tanzania 80.7%; Zambia 58.5%). Across all three countries, the mean age for men was higher compared to women and more men had completed secondary education or higher. The most common occupation for men was agriculture in Malawi 39.5%; Tanzania 56.7%; Zambia 56.3%.

Many Tanzanian (62.4%) and Zambian (53.6%) women believed being beaten by their husbands was justified, but this was not a commonly reported belief among Malawian women (16.0%). More than half of Malawian (56.9%) and Zambian (53.5%) mothers reported being afraid of their husbands at least sometimes. Men rarely had been physically hurt by their wife or partner (Malawi 3.9%; Tanzania 1.8%; Zambia 6.3%), although it is important to note this question was self-reported by the wives/partners and the men were not asked directly.

### Breastfeeding

Early initiation of breastfeeding and EBF varied across countries while continued breastfeeding practices were similar (Table 2). While 77.6% of Malawian mothers initiated breastfeeding within 1 h of birth, this percentage was lower in Tanzania (53.6%) and Zambia (67.7%). Just over half (55.0%) of Tanzanian mothers exclusively breastfed but exclusive breastfeeding was higher in Malawi (60.1%) and Zambia (70.6%). Continued breastfeeding until the child’s first birthday was high in all countries: Malawi 92.2%, Tanzania 93.4%, and Zambia 95.0%.

**Table 2:** Breastfeeding Indicators and Maternal Experience of Intimate Partner Violence (IPV) in Malawi, Tanzania, & Zambia

	Malawi		Tanzania		Zambia	
	%	n	%	n	%	n
**IYCF**						
Early Initiation of Breastfeeding	77.6	1456/1878	53.6	1705/3184	67.7	3147/3879
EBF at 6 months	60.1	256/426	55.0	396/720	70.6	607/860
Continued Breastfeeding at 1 year	92.2	302/328	93.4	516/553	95.0	631/664
**IPV**						
Experienced Any IPV^a^	73.9	1389/1878	79.1	2519/3184	78.9	3063/3879
**Form of IPV**		1878		3184		3879
Physical Violence	22.8	428	38.5	1228	37.5	1453
Emotional Violence	25.8	484	35.8	1141	21.8	846
Sexual Violence	18.3	343	13.0	414	16.5	641
Controlling Behaviors	69.1	1297	70.2	2234	73.6	2854
**Cumulative Forms of IPV**^a^		1878		3184		3879
0	26.0	489	20.9	665	21.0	816
1	38.0	715	49.7	1582	57.2	2218
2	20.6	386	20.6	654	2.6	100
3	8.3	156	0.7	25	11.8	460
4	7.1	133	8.1	257	7.4	285
**Frequency of IPV**						
**Experienced Any IPV**^b^		1878		3184		3879
Never	60.9	1143	50.9	1621	54.3	2106
Yes but not in last 12 months	7.6	143	34.5	1097	20.9	810
Often or sometimes	31.5	592	14.6	466	24.8	963
**Physical Violence**		1878		3184		3879
Never	77.2	1450	61.4	1956	62.5	2426
Yes but not in last 12 months	8.6	161	27.2	864	17.5	678
Often or sometimes	14.2	267	11.4	364	20.0	775
**Emotional Violence**		1878		3184		3879
Never	74.2	1394	64.2	2043	78.2	3033
Yes but not in last 12 months	4.8	90	29.0	923	5.4	209
Often or sometimes	21.0	394	6.8	218	16.4	637
**Sexual Violence**		1878		3184		3879
Never	81.7	1535	87.0	2770	83.5	3238
Yes but not in last 12 months	3.9	73	10.9	347	3.8	149
Often or sometimes	14.4	270	2.1	67	12.7	492

^a^Includes controlling behaviors

^b^Does not include controlling behaviors

### Intimate partner violence

Intimate partner violence was similar across countries (Table 2). Many of the mothers in all three countries reported experiences of IPV in Tanzania (79.1%), Zambia (78.9%), and Malawi (73.9%). In all three countries, controlling behaviors were the most commonly reported IPV and sexual IPV was the least commonly reported. Among women who reported IPV, many experienced only one form. However, in both Malawi and Tanzania, 20.6% reported experiencing two forms of IPV, and in Zambia 11.8% experienced three forms of intimate partner violence. The frequency of the different types of IPV varied across countries. A higher percentage of Malawian and Zambian mothers reported experiencing IPV often or sometimes compared to mothers in Tanzania.

### Intimate partner violence and delayed initiation of breastfeeding

Table 3 highlights the notable similarities for associations between IPV and early initiation of breastfeeding in Malawi, Tanzania, and Zambia. In all three countries, mothers who experienced sexual IPV were more likely to delay breastfeeding in Malawi [AOR 1.55 (1.14, 2.10), *p* < 0.01]; Tanzania [AOR 1.30 (1.04, 1.62), *p* < 0.05]; and Zambia [AOR 1.28 (1.06, 1.54), *p* < 0.05]. Also, in Zambia, mothers whose partners exhibited controlling behaviors were more likely to delay breastfeeding [AOR 1.28 (1.07, 1.53), *p* < 0.01]. In bivariate analyses, women in all three countries who experienced emotional IPV were more likely not to initiate early breastfeeding. The association in Zambia did not reach significance [AOR 1.16 (0.99, 1.02), *p* = 0.05] in the adjusted model, but the association was significant for Malawi [AOR 1.37 (1.05, 1.80), *p* < 0.05] and Tanzania [AOR 1.43 (1.22, 1.68), *p* < 0.0001].

**Table 3:** Adjusted associations between maternal experience of intimate partner violence (IPV) and delayed initiation of breastfeeding

	Delayed Initiation of Breastfeeding		
Forms of IPV	Malawi (***n*** = 1878)	Tanzania (***n*** = 3184)	Zambia (***n*** = 3879)
Physical – ever	**–**	1.44 (1.24,1.69)****	1.13 (0.98,1.30)
Emotional – ever	1.37 (1.05,1.80)*	1.43 (1.22,1.68)****	1.16 (0.99,1.02)
Sexual – ever	1.55 (1.14,2.10)**	1.30 (1.04,1.62)*	1.28 (1.06,1.54)*
Controlling Behaviors – ever	–	–	1.28 (1.07,1.53)**
**Frequency of IPV**			
**Physical**			
Never	–	1	1
Yes but not in last 12 months	–	1.38 (1.16,1.65)***	1.11 (0.96,1.37)
Often or sometimes	–	1.55 (1.23,1.97)***	1.14 (0.91,1.34)
**Emotional**			
Never	1	1	1
Yes but not in last 12 months	1.56 (0.90,2.68)	1.43 (1.20,1.69)***	1.35 (0.98,1.33)
Often or sometimes	1.34 (0.99,1.79)	1.45 (1.08,1.95)*	1.10 (0.92,1.84)
**Sexual**			
Never	1	–	1
Yes but not in last 12 months	1.83 (0.98,3.42)	–	1.55 (1.06,2.27)*
Often or sometimes	1.49 (1.08,2.06)*		1.22 (1.01,1.49)*

Models adjusted for strata, cluster, child sex, maternal age, maternal occupation, maternal wealth index, residence (urban or rural), maternal education, maternal literacy, maternal exposure to radio, newspaper, or TV, type of delivery (C-section or vaginal), delivery location, birth spacing, and antenatal visits. (−) indicates not significant in bivariate analyses

Results expressed as adjusted odds ratio AOR (95% CI); *p-value < 0.05, **p-value< 0.01, ***p-value< 0.001, ****p-value< 0.0001

In addition to analyzing the four different forms of IPV experienced by mothers, the frequency of IPV experiences was investigated. In both Malawi and Zambia, mothers who experienced sexual violence often or sometimes were more likely than other mothers to delay breastfeeding (Malawi [AOR 1.49 (1.08, 2.06), *p* < 0.05; Zambia [AOR 1.22 (1.01, 1.49), *p* < 0.05]). Tanzanian mothers who experienced physical IPV [AOR 1.55 (1.23, 1.97), *p* < 0.001], or emotional IPV [AOR 1.45 (1.08, 1.95), *p* < 0.05] often or sometimes were more likely not to have initiated early breastfeeding. Furthermore, Tanzanian women who experienced emotional IPV [AOR 1.43 (1.20, 1.69), *p* < 0.001], or physical IPV [AOR 1.38 (1.16, 1.65), *p* < 0.001] more than 1 year ago also were more likely not to have initiated early breastfeeding.

### Intimate partner violence and non-exclusive breastfeeding

Associations between IPV and EBF generally differed across countries (Table 4). The only similarity was that women in both Malawi and Zambia who experienced sexual IPV had increased odds of not exclusively breastfeeding (Malawi [AOR 1.90 (1.05, 3.45), *p* < 0.05]; Zambia [AOR 1.75 (1.15, 2.67), *p* < 0.01]). Additionally, in Zambia, infants born to mothers who experienced physical IPV [AOR 1.82 (1.31, 2.51), *p* < 0.001] or emotional IPV [AOR 1.72 (1.18, 2.50), *p* < 0.01] were more likely not to be exclusively breastfed. In bivariate analysis, mothers in Tanzania who experienced emotional IPV often or sometimes were more likely not to have exclusively breastfeed, but this did not remain significant in the adjusted models. On the contrary, Tanzanian women who experienced emotional IPV more than 1 year prior to the survey were more likely to have exclusively breastfed [AOR 0.38 (0.18, 0.78), *p* < 0.01] compared to women who reported no experiences of intimate partner violence.

**Table 4:** Adjusted associations between maternal experience of intimate partner violence (IPV) and non-exclusive breastfeeding

	Non-Exclusive Breastfeeding		
Forms of IPV	Malawi (***n*** = 426)	Tanzania (***n*** = 720)	Zambia (***n*** = 860)
Physical – ever	**–**	–	1.82 (1.31,2.51)***
Emotional – ever	–	–	1.72 (1.18,2.50)**
Sexual – ever	1.90 (1.05,3.45)*	–	1.75 (1.15,2.67)**
Controlling Behaviors – ever	–	1.38 (0.96,1.99)	–
**Frequency of IPV**			
**Physical**			
Never	1	1	1
Yes but not in last 12 months	1.92 (0.99,3.72)	0.60 (0.35,1.02)	1.52 (0.99,2.33)
Often or sometimes	1.04 (0.46,2.32)	1.43 (0.98,2.09)	2.11 (1.42,3.14)***
**Emotional**			
Never	–	1	1
Yes but not in last 12 months	–	0.38 (0.18,0.78)**	1.64 (0.83,3.26)
Often or sometimes	–	1.07 (0.73,1.55)	1.71 (1.13,2.60)*
**Sexual**			
Never	1	–	1
Yes but not in last 12 months	1.34 (0.37,4.81)	–	1.10 (0.38,3.21)
Often or sometimes	2.07 (1.08,3.96)*	–	1.89 (1.21,2.95)**

Models adjusted for strata, cluster, child sex, maternal age, maternal occupation, maternal wealth index, residence (urban or rural), maternal education, maternal literacy, maternal exposure to radio, newspaper, or TV, type of delivery (C-section or vaginal), delivery location, birth spacing, and antenatal visits. (−) indicates not significant in bivariate analyses

Results expressed as adjusted odds ratio AOR (95% CI); *p-value < 0.05, **p-value< 0.01, ***p-value< 0.001

### Intimate partner violence and cessation of breastfeeding prior to 1 year

Evidence signifying associations between IPV and continued breastfeeding until the child’s first birthday were lacking (Table 5). The only significant finding was that Tanzanian mothers who often or sometimes experienced emotional IPV were two times more likely not to continue breastfeeding for a full year [AOR 2.23 (2.09, 4.57), *p* < 0.05]. There were no significant associations between IPV and continued breastfeeding among Malawian and Zambian mothers.

**Table 5:** Adjusted associations between maternal experience of intimate partner violence (IPV) and cessation of breastfeeding

	Cessation of Breastfeeding Prior to One Year		
Forms of IPV	Malawi (***n*** = 335)	Tanzania (***n*** = 553)	Zambia (***n*** = 666)
Physical – ever	**–**	–	–
Emotional – ever	**–**	1.89 (0.94,3.77)	–
Sexual – ever	–	–	–
Controlling Behaviors – ever	–	–	–
**Frequency of IPV**			
**Physical**			
Never	–	–	–
Yes but not in last 12 months	–	–	–
Often or sometimes	–	–	–
**Emotional**			
Never	–	1	–
Yes but not in last 12 months	–	0.70 (0.14,3.55)	–
Often or sometimes	–	2.23 (1.09,4.57)*	–
**Sexual**			
Never	–	–	–
Yes but not in last 12 months	–	–	–
Often or sometimes	–	–	–

Adjusted for strata, cluster, child sex, maternal age, maternal occupation, maternal wealth index, residence (urban or rural), maternal education, maternal literacy, maternal exposure to radio, newspaper, or TV, type of delivery (C-section or vaginal), delivery location, birth spacing, and antenatal visits. (−) indicates not significant in bivariate analyses

Results expressed as adjusted odds ratio AOR (95% CI); *p-value < 0.05

## Discussion

This study used nationally representative data from Malawi, Tanzania, and Zambia to investigate associations between physical, sexual, and emotional violence, as well as controlling behaviors, with early initiation of breastfeeding, EBF, and continued breastfeeding. Additionally, associations between frequency of IPV, whether often/sometimes or more than one-year prior, were analyzed for each breastfeeding indicator. The major finding was that maternal experience of various types of IPV was associated with suboptimal breastfeeding practices in all three countries.

### Intimate partner violence and delayed initiation of breastfeeding

In all three countries, maternal experience of any IPV was negatively associated with early initiation of breastfeeding which is consistent with the deficit hypothesis. Furthermore, experiencing emotional IPV specifically was associated with delayed initiation of breastfeeding in Malawi and Tanzania, which conflicts with previous findings from DHS analyses that showed no significance between emotional IPV and early initiation of breastfeeding in these countries [20]. However, similar to our findings, maternal experience of emotional IPV was associated with breastfeeding avoidance and higher odds of delayed initiation of breastfeeding in both Spain and Kenya [20, 23]. Furthermore, a population-based study including pooled results from 51 low-income and middle-income countries found maternal lifetime experience of emotional violence was associated with lower odds of early initiation of breastfeeding [14].

Because the few studies that have analyzed emotional or psychological IPV have been cross-sectional and cannot determine casual pathways, the mechanism of the association remains unclear. Experience of IPV has been associated with poor self-esteem [24] and both low self-efficacy and low confidence have been identified as barriers to optimal breastfeeding practices [25, 26]. That delayed initiation of breastfeeding among Tanzanian mothers was associated both with those who experienced emotional IPV often or sometimes and those who had experienced emotional IPV in the past illustrates the potential long-term negative influence of emotional IPV on breastfeeding. However, the proposed link between experience of IPV, low self-efficacy, and delayed initiation is only one plausible pathway. Future studies may identify other ways by which experiencing emotional IPV leads to delayed initiation of breastfeeding.

Additionally, mothers in all three countries who experienced sexual IPV were more likely to delay breastfeeding. Results from Malawi and Tanzania [20] showed no significant association between IPV and delayed breastfeeding initiation. However, our results are consistent with findings in India [18] and Zambia [20]. The negative impact of experiencing sexual IPV on mothers may contribute to the barriers and challenges of early initiation of breastfeeding. One study has suggested that maternal experience of sexual IPV may lead mothers to develop negative associations with breastfeeding [27]. Furthermore, it has been hypothesized that any previous experience of sexual IPV may increase a mother’s cortisol levels [12] which has been linked to delayed onset of milk production up to 3 or 4 days postpartum [28]. Due to the limited available data on sexual IPV and early initiation of breastfeeding, investigation of additional possible mechanisms of how experiencing sexual IPV negatively impacts early initiation of breastfeeding would be beneficial.

In Zambia, mothers whose partners had controlling behaviors were more likely to delay the initiation of breastfeeding. Perhaps the partner’s controlling behaviors diminished women’s empowerment, which has been identified as a key factor in optimal breastfeeding practices [21]. Because this study is the first to identify controlling behaviors as negatively associated with breastfeeding, future research should consider analyzing which specific controlling behaviors are most detrimental for breastfeeding practices. Furthermore, potential mediating factors should be identified to help women overcome the negative influence of controlling behaviors on early initiation of breastfeeding.

In addition to the negative association across countries between early initiation of breastfeeding and sexual or emotional IPV, and controlling behaviors, physical IPV was also negatively associated with early initiation of breastfeeding in Tanzanian women regardless of whether the IPV was recent or in the past. Even experiencing physical IPV more than 1 year prior to the study may negatively impact breastfeeding as IPV is known to have long-term negative effects [11]. Similar to sexual IPV, physical IPV can lead to increased maternal cortisol [28]. Additionally, a mother’s body, including her mammary glands, may have sustained physical damage as a result of physical IPV [11] thus interfering with early initiation of breastfeeding.

### Intimate partner violence and non-exclusive breastfeeding

Associations between maternal experience of IPV and non-exclusive breastfeeding generally varied across countries. Similar to findings in other countries [14, 17, 18, 29], children born to Zambian mothers who reported physical IPV were more likely to receive foods or fluids other than breastmilk before 6 months of age. Likewise, both Malawian and Zambian mothers who experienced sexual violence had higher odds of not exclusively breastfeeding.

Contrary to all other findings of this study, Tanzanian women who experienced emotional IPV more than 1 year prior to the survey were more likely to have exclusively breastfed. These results are consistent with the compensatory hypothesis. Since the reported IPV occurred more than 1 year prior to the study, it’s possible that these mothers were able to receive help to overcome the possible negative effects IPV might have had on their breastfeeding practices. The results in Tanzania differed from Zambian mothers who, if they had experienced emotional violence often or sometimes, were more likely to give foods or other liquids to the infant before 6 months of age. These contradictory results between countries suggest there may be other mediating factors that are country specific, such as breastfeeding support for women or psychosocial counseling resources that mediate the influence of IPV on breastfeeding.

However, further investigation is warranted to better understand how prior experience of IPV led mothers in Tanzania to be more likely to exclusively breastfeed. Understanding Tanzanian mothers’ intention and choice to exclusively breastfeed despite their experience of IPV could be beneficial in designing interventions that provide breastfeeding support for other mothers who experience intimate partner violence. Future studies may consider exploring the use of breastfeeding counselling or other culturally-specific social support interventions as ways to mitigate the negative influence of IPV on EBF in Malawi and Zambia.

### Intimate partner violence and cessation of breastfeeding prior to 1 year

To the best of our knowledge, this study was the first to analyze the associations between IPV and continued breastfeeding. Intimate partner violence was associated with cessation of breastfeeding in Tanzania among mothers who experienced emotional IPV often or sometimes. These mothers were two times more likely to stop breastfeeding before the child’s first birthday compared to mothers with no reported experience of intimate partner violence. Frequent experience of emotional IPV has been shown to be associated with poor self-esteem [24]. However, these findings were not consistent in Malawi and Zambia; thus, the association between maternal experience of IPV and continued breastfeeding remains unclear. In this context, however, it is important to consider that overall adherence to continued breastfeeding recommendations was high in all three countries.

### Recommendations

National strategic plans are prioritizing improvements in breastfeeding in Malawi, Tanzania, and Zambia [30–32]. In support of these efforts, screening women for IPV during antenatal care and providing access to services may at least partially mitigate negative effects of IPV, and therefore may be imperative to support breastfeeding mothers. While the WHO (2016) recommends that screening for IPV be conducted during antenatal visits [33], the feasibility of this practice in each country must be addressed. In Tanzania, screening for IPV among women attending outpatient clinics has been initiated [34], but future studies may need to consider the feasibility of IPV screening during antenatal care in these countries.

Furthermore, public health activities that support prevention of sexual IPV, as well as adequate screening of sexual IPV during antenatal care, may allow mothers to receive support and guidance on early lactation management. In addition to screening for IPV during antenatal care, women also should be provided with appropriate physical exams to be aware of any possible bodily damage that may hinder her ability to breastfeed. Because breastfeeding counseling [35] and social support [36] have been effective facilitators of breastfeeding in other countries among women who experience of IPV, both of these interventions may mediate the association between maternal experience of IPV and optimal breastfeeding in Malawi, Tanzania, and Zambia.

### Limitations

Despite its strengths, the study had limitations. The use of cross-sectional data does not provide insight on causality and EBF was only queried for the previous 24 h and therefore, may not be reflective of the infant’s first 6 months of life. Additionally, the CTS-2 has not been validated in Malawi, Tanzania, and Zambia, and cross-cultural reliability has not yet been established in these countries. Furthermore, some maternal experiences of IPV may have remained undisclosed, resulting in underestimation of IPV, while social desirability bias may have resulted in overestimation of breastfeeding adherence. Measures of emotional/psychological IPV have varied across studies which is a barrier to comparing study results. Lastly, measuring IPV alone does not account for other violent experiences a mother may have encountered which also may impact breastfeeding.

## Conclusions

Early initiation of breastfeeding and exclusive breastfeeding practices remains below the WHO targets for 2030 [4] in Malawi, Tanzania, and Zambia. Each type of IPV was negatively associated with optimal breastfeeding practices in at least one country and at least two forms of IPV were associated with a higher likelihood of delayed breastfeeding in each country. Therefore, for interventions to be effective in improving breastfeeding practices, the potential negative impact of IPV on breastfeeding outcomes must be considered. Policies to implement IPV screening during antenatal and postnatal care and referral of mothers for culturally sensitive assistance and care may improve breastfeeding practices. The importance of breastfeeding remains a constant focus for public health programs in Africa. Therefore, both raising awareness on the negative impact of IPV on maternal, infant, and child health, as well as supporting programs that reduce IPV are crucial.

## Data Availability

The data analyzed can be accessed upon approval from the DHS Program at https://www.dhsprogram.com/data.

## Abbreviations

AOR: Adjusted odds ratio
C-section: Caesarean section
CI: Confidence interval
CTS-2: Revised Conflict Tactics Scale
DHS: Demographic and Health Survey
EBF: Exclusive breastfeeding
IPV: Intimate partner violence
IYCF: Infant and Young Child Feeding
SAS: Statistical Analysis System
TV: Television
VIF: Variance inflation factor
WHO: World Health Organization
